# WO_3_ in suit embed into MIL-101 for enhancement charge carrier separation of photocatalyst

**DOI:** 10.1038/s41598-019-41374-z

**Published:** 2019-03-19

**Authors:** Linjuan Wang, Ling Zan

**Affiliations:** College of Chemistry and Molecular Science, Wuhan University, Wuhan, 430072 P. R. China

## Abstract

Compositing nanoparticles photo-catalyst with enormous surface areas metal–organic framework (MOF) will greatly improve photocatalytic performances. Herein, WO_3_ nanoparticles are partly embedded into pores of MIL-101 or only supported on the outside of representative MIL-101, which were defined as embedded structure WO_3_@MIL-101@WO_3_ and coating structure WO_3_&MIL-101 respectively. Different pH, concentration and loading percentage were researched. XRD, TEM and BET were carried to analyze the composites. Compared with the pristine WO_3_, all WO_3_ loaded MOF nanocomposites exhibited remarkable enhancing for the efficiency of photocatalytic degradation methylene blue under visible light. Their activity of the same loading percentage WO_3_ in embedded structure and coating structure have increased for 9 and 3 times respectively compared with pure WO_3_. The WO_3_@MIL-101@WO_3_ has 3 times higher efficiency than WO_3_&MIL-101, because the shorter electron-transport distance can make a contribution to electron–hole separation. The further mechanism involved has been investigated by radical quantify experiment, XPS and photoluminescence spectroscopy.

## Introduction

Semiconductor nanoparticles are considered to be a superior photo catalyst for completely eliminating hazardous wastes and toxic contaminants caused by urbanization and industrialization because of their high photocatalytic activity and strong quantum-size effect^1,2^. However, the properties of some semiconductors are ultraviolet light absorbing consisting only 4% among the whole solar energy^3–6^, and have a tendency to aggregate, the two defects limited the photo catalyst’s universal usage. So, minimizing the particle size and exploring wide light spectrum catalyst have been a hot topic in the photo catalyst research. One of visible light absorbing photo catalyst WO_3_ has become a fiercely debated material owing to the following advantages: (1) it is very stable, non-toxic and economic to synthesize which can be recycled and commonly used^7–9^; (2) it can absorb visible spectrum light consisting 43% solar energy with a narrow band gap (2.7 eV)^10–12^; (3) suitable band alignment with a relatively positive valence band position allows its strong oxidation degradation effect.

However, pure WO_3_ has a low photocatalytic performance. Some methods have been tried to solve these problems, such as synthesize WO_3_ quantum dots to minimize the particle size^13^, loading WO_3_ with Pt nanoparticles to enhance the electrons transferring^14–21^. Up to now, the problems cannot be solved thoroughly. Some MOFs have appeared as potential candidates for photo catalysis because of their high surface areas^22–27^, tunable porosity^28,29^, crystalline open structures and multi-functionalities^30–32^. Due to their enormous inner surface, total surface area as much as 2000 times larger compared with silicon and grapheme which are usually used as carrier material in the catalyst field according to previously reports. As reported, MOFs have been found to be ideal materials for dispersing metal and semiconductor nanoparticles because their high surface area can avoid the aggregation of nanoparticles^27^. Besides, the large specific surface area of MOFs also can supply a plenty of active adsorption sites and photocatalytic reaction centers, which would enhance the photocatalytic properties^27,33,34^. The special structure of MOFs composited with organic ligands and metal ions allow the metal center acting as the shallow electron trap during the process of electrons transportation^35,36^. The synergistic effect between MOFs and semiconductor can promote the charge separation and enhance the photocatalytic activity^27,37^.

Particularly, many researchers have reported on MOFs based hetero structure photo-catalyst^38–40^. Au@CdS@MIL-101^27^, Co_x_@MIL-101^41^, Pd@MIL-101^42^ and Pt@MIL-101 (Cr)^43^ have been investigated. But most of which are loaded photo-catalyst on surface of MIL-101^27^, the large inner area of the pores cannot be used fully.

In this paper, we synthesized WO_3_ embed into the pores of MIL-101, and researched its photocatalytic properties, then compared with pure WO_3_ and only coating outside MIL-101. Different pH, concentration and loading percentage were researched to boost the photocatalytic activity. Compared with WO_3_&MIL-101 and pure WO_3_, the photocatalytic efficiency of the embedded structure has improved 9 and 3 times respectively, and the pore size distribution and adsorption-desorption isotherm demonstrated that the WO_3_ nanoparticles have embedded into the pores of MIL-101. The mechanism has been studied by trapped the active species hydroxyl radicals.

## Experimental

### Reagents and chemicals

All the chemicals were purchased from commercial sources and were utilized without further purification. Sodium tungstate and Terephthalic acid were applied by Aladdin Reagent Co. Ltd. Hydrochloricacid (HCl, 37%), Hydrofluoric acid (HF, 49%), Hydrogen peroxide (H_2_O_2_, 30%), Dimethyl formamide (DMF > 99.8%), Anhydrous ethanol, Chromic nitrate, were obtained from Sinopharm Chemical Reagent Co. Ltd. China. The solvent is water and is Ultra purified (18 Mπ·cm).

### Synthesis of MIL-101

The MIL-101 was prepared via a hydrothermal method according to literature with slight modification^27^. Commonly, 0.8 g Cr(NO_3_)_3_·9H_2_O, 100 μL hydrofluoric acid (40%), 800 mg p-phthalic acid were put in 12.5 mL H_2_O solvent. The system was ultrasonic under room temperature continued 30 min then transformed into 25 mL autoclave and maintained at 220 °C for 8 h. After cooling to room temperature, the resultant solid was isolated by filtration and rinsed with DMF and ethanol several times to remove remained substances, treated solvothermal with ethanol at 100 °C for 12 h, collected by filtration, dried at 80 °C, vacuum dried at 150 °C and then stored for further use.

### Synthesis of WO_3_@MIL-101@WO_3_

Different sodium tungstate quality, pH and concentration were investigated, the different condition were listed as Table S1. Typically, 320 μL hydrochloric acid which was diluted to 5 mL with water, then 10 mg sodium tungstate was dissolved in 40 mL water was added. After adding 100 μL hydrogen peroxide, the mixture was stirred for 30 min. Then 100 mg MIL-101 was added to the solution, stirring for 24 h under room temperature. Then the mixture was stirring and heated with oil bath by stepwise warming method. After temperature raised to 30 °C, it was kept at 30 °C for 1 h. Then raise the temperature to 45 °C and kept it for 1 h. After that it was raised to 60 °C and kept for 3 h, the temperature was raised to 120 °C until the water was dried up. The substance was washed with water and ethanol several times.

### Synthesis of WO_3_&MIL-101

In order to deposit WO_3_ completely outside of MIL-101, direct settlement method is used and with MIL-101 not being degassed. With the same steps to prepare WO_3_ precursor, then 100 mg not degassed MIL-101 was added to the solution, stirring for 30 min, the temperature was raised to 120 °C for the water drying up. The substance was washed with water and ethanol several times.

### Characterization

The crystal structure of the prepared samples were characterized by a Bruker D8 Advance X-ray diffractometer with Ni-filtered Cu KαIrradiation (λ = 0.15406 nm) under 40 kV and 40 mA. XPS diffraction patterns were carried out by an AXIS-His spectrometer (Kratos Corporation) with a Mg Kα X-ray source, and the spectra were adjusted to the C 1 s peak at 284.8 eV. The shape and size of the nanocomposites were characterized by a JEOL JEM-6700F field emission scanning electron microscope with an accelerating voltage of 20 kV, respectively. TEM and HRTEM images were obtained under a JEM-2100 transmission electron microscope with an accelerating voltage of 20 kV. The surface area and the pore size distribution were measured on Quantachrome Autosorb-IQ sorption system at 77 K. Optical absorption properties (DRS) were detected under a Shimadzu UV-3600 spectrometer with a reference of BaSO_4_. The photoluminescence (PL) emission spectra of samples were observed on a Hitachi F-4500 luminescence spectrometer.

### Photocatalytic Activity Test

The photocatalytic degradation performance of Methylene Blue (MB) test was carried out under visible light irradiation^44^. A xenon lamp (300 W) with visual light filter was dispersed in an aqueous solution (50 mL) containing 30 mg/L MB dye by ultrasonic treatment for 5 min and maintained stirring for 30 min. Then, the solution was transferred to a quartz reaction vessel and agitated for some time. A liquid (5 mL) was sampled at scheduled irradiation time and the suspended catalyst were eliminated by centrifugation under 8000 rpm for 5 min. The UV-Visible absorption spectrum of the solution was carried out with a UV-Visible absorption spectrum of the solution was carried out with a UV-Vis spectrophotometer (UV-3600). The percentage of degradation was defined as −ln (*C/C*_0_), herein, *C*_0_ refers the absorption (*λ*_max_ = 664 nm) of MB solution prior irradiation and *C* indicates the absorption of MB solution at each irradiated time interval.

### Active Species Trapping and Superoxide Radical Quantification Experiments

For detecting the active species during photocatalytic reactivity^45^, hydroxyl radicals (·OH), the superoxide radical (O_2_^−^·), and holes (h^+^) were trapped by adding 2.0 mM (according to the reaction system) IPA^46^ (a quencher of ·OH), AgNO_3_^47^ (a quencher of O_2_^−^·), and TEOA^48^ a quencher of h^+^ respectively. The method was similar to the former photocatalytic activity test^45^. TA (5 × 10^−4^ M in a 2 × 10^−3^ M NaOH solution), which reacts readily with ·OH generating from WO_3_&MIL-101 and WO_3_@MIL-101@WO_3_. The production of ·OH was quantitatively analyzed by detecting the concentration of 2-hydroxyterephthalic acid (fluorescence peak at about 425 nm by excitation with the wavelength of 315 nm) with Shi-madzu spectro fluorophotometer (RF-5301 pc) after centrifugation^49^. The method was similar to the former photocatalytic activity test, with TA replacing the MB^48^.

## Results and Discussion

### Reaction Process Illustration

**Figure 1:**
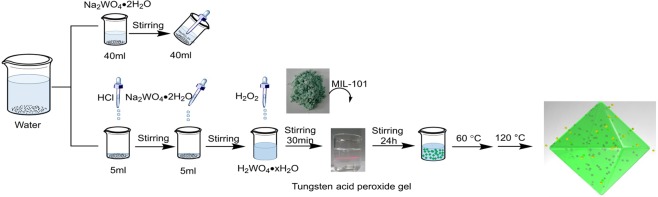
The reaction process of the formation of embedded structure WO_3_@MIL-101@WO_3_.

As shown in Fig. 1, the WO_3_@MIL-101@WO_3_ hetero-structure were synthesized by low temperature H_2_O_2_ assistant sol-gel method. The process of adding H_2_O_2_ was very important for the formation of peroxo-tungstate gel precursor, obvious Tyndall effect can be observed. Stirring for 24 h giving enough time for the slow kinetic reaction process of MIL-101 dipping into peroxo tungstate gel. The loading percentage, pH and concentration influencing the properties of precursor were also researched listed in Table S1. The resultant WO_3_&MIL-101 and WO_3_@MIL-101@WO_3_ samples have been well characterized by various techniques. The actual loading percentages tungsten (5%-15%) at various precursor concentrations determined by atomic absorption spectrum (AAS) method matches well with the theoretical loading (5.27–15.5%), as shown in Table S2, indicating that the H_2_O_2_ assistant sol-gel method is effective in loading WO_3_ into MIL-101.

### Structure, composition, and microstructure

**Figure 2:**
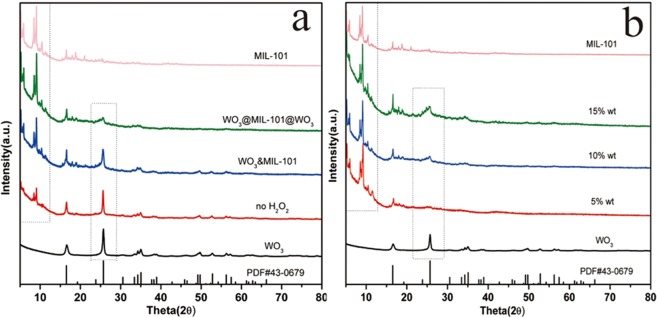
(**a**) XRD patterns of MIL-101, WO_3_@MIL-101@WO_3_, WO_3_&MIL-101, no hydrogen H_2_O_2_ and WO_3_. (**b**) The XRD patterns of samples with different loading percentage.

As shown in Fig. 2a, the crystal structure of MIL-101 is in good agreement with the literature reported^41^, demonstrating the formation of MIL-101 with ultrapure and good crystallinity. After loading WO_3_, the characteristic XRD peaks of MIL-101 in all samples are maintained, demonstrating the treatment did not have the damage on the crystal structure of MIL-101. The weaker peaks of WO_3_@MIL-101@WO_3_ than WO_3_&MIL-101 should be due to a part of WO_3_ have been embedded into pores of MIL-101 which resulted in small particle size. The patterns of different loading percentage were shown in Fig. 2b, as the loading percentage increased the intensity of the characteristic peaks increases. Different pH and concentration also show a significant influence on the intensity of MIL-101 shown in Fig. S1a,b.

**Figure 3:**
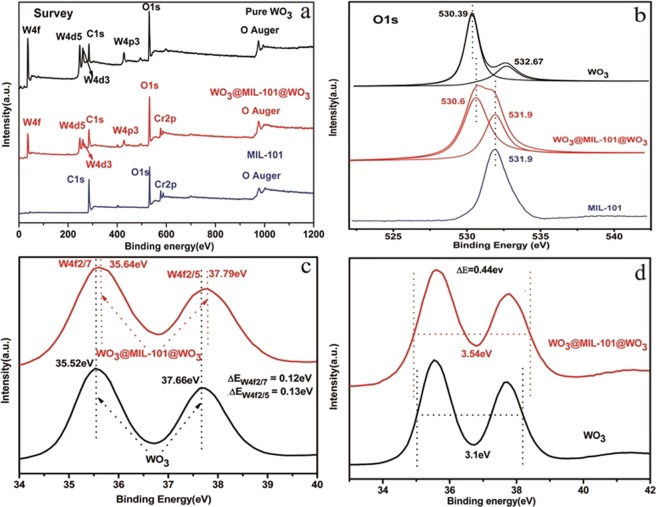
XPS. (**a**) The survey pattern of WO_3_, WO_3_@MIL-101@WO_3_, and MIL-101. (**b**) The shift and changed of O high resolution pattern of WO_3_, MIL-101, and WO_3_@MIL-101@WO_3_. (**c**) The shift of W element in WO_3_@MIL-101@WO_3_ and WO_3_, (**d**) The enlarged of half band width of W element in WO_3_@MIL-101@WO_3_ compared with WO_3_.

The survey pattern of WO_3_, WO_3_@MIL-101@WO_3_, and MIL-101 was shown in Fig. 3a.The high resolution XPS of O element (Fig. 3b) show the position 530.6 eV for WO_3_@MIL-101@WO_3_ which has right shift 0.21 eV compared with WO_3_, the absorbed oxygen at 532.67 eV was disappeared, indicating the O element has some changes in WO_3_@MIL-101@WO_3_ compared with pure WO_3_. Figure 3c,d show the binding energy shift and half band width changes, W_4f2/7_ and W_4f2/5_ have right shift 0.12 eV and 0.13 eV, respectively. The half band width of WO_3_@MIL-101@WO_3_ has been widen 0.44 eV compared with WO_3_, these results all show that W element has a good attachment with the linkages of MIL-101, there are interactions between WO_3_ and MIL-101.

In order to further detect the relation of WO_3_ and MIL-101, DLS method was adapted. In Table S3, The zeta potential of WO_3_, WO_3_&MIL-101,WO_3_@MIL-101@WO_3_ and MIL-101 are −34.1 mV, −8.69 mV, −6.04 mV and 34.5 mV respectively. The result for WO_3_&MIL-101 (−8.69 mV) and WO_3_@MIL-101@WO_3_ (−6.04 mV) shows electrostatic attraction between MIL-101 and oppositely charged WO_3_. A close interaction of the WO_3_&MIL-101 and WO_3_@MIL-101@WO_3_ composite can be achieved with the electrostatic attraction. The more negative potential of WO_3_&MIL-101 than WO_3_@MIL-101@WO_3_ indicates there exist more WO_3_ nanoparticles on the surface of MIL-101.

**Figure 4:**
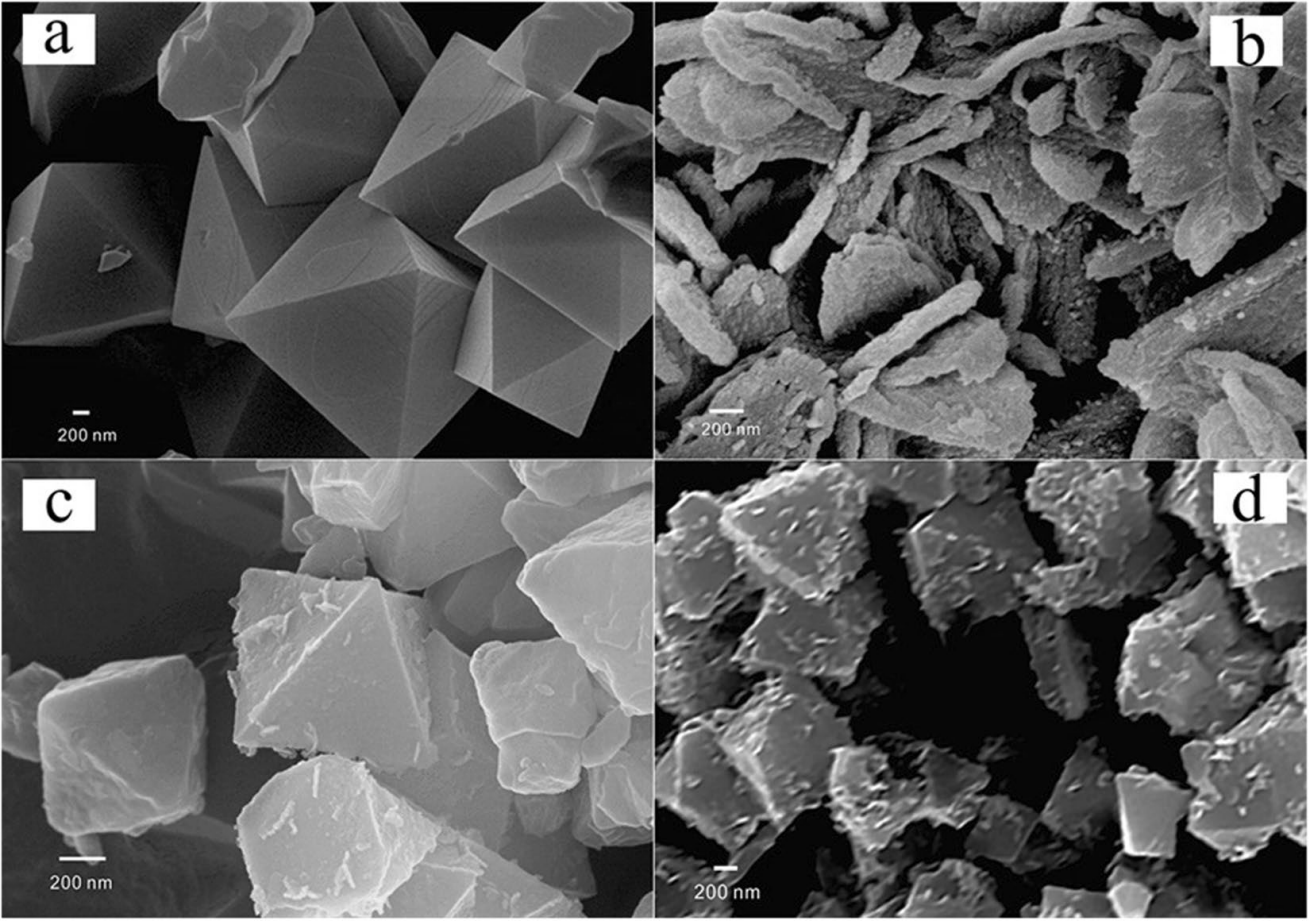
SEM images of (**a**) MIL-101, (**b**) WO_3_, (**c**) (10%) WO_3_@MIL-101@WO_3_, (**d**) (10%) WO_3_&MIL-101.

As shown in Fig. 4a, octahedral structure with smooth surface of MIL-101 have the size of 400 nm–600 nm^27^. As shown in Fig. 4b, the size of pure WO_3_ nanosheets are about 50 nm in thickness and 400 nm in width. After loading WO_3_, the surface of MIL-101 became rough and coating a slice WO_3_ on the surface of MIL-101 for WO_3_@MIL-101@WO_3_. For WO_3_&MIL-101, WO_3_ particles growth and there are intensity aggregating together with each other. The average particle size is about 40 nm shown as Fig. S2d. For the exploration experiment, we investigated the WO_3_@MIL-101@WO_3_ samples with different WO_3_ loading proportion (5–15%), different pH, different concentration and not adding hydrogen peroxide and the SEM results are displayed in Figs S2 and S3, and S4. As can be seen from Fig. S2, WO_3_ were thinly well coating on MIL-101 much like the morphology of WO_3_@MIL-101@WO_3_, as the loading proportion increasing, the amount of WO_3_ slice increase. Figure S2c show no hydrogen peroxide added in the solution, WO_3_ nanoparticles have grown much larger, indicating that the adding hydrogen peroxide can change the state of peroxo tungstate precursor gel, which is very important for WO_3_ embed into the pores of MIL-101. As can be seen Figs S3 and S4 in all WO_3_@MIL-101@WO_3_ samples, WO_3_ are all well slice coating outside MIL-101, with pH and concentration changes, the state of WO_3_ have some difference.

**Figure 5:**
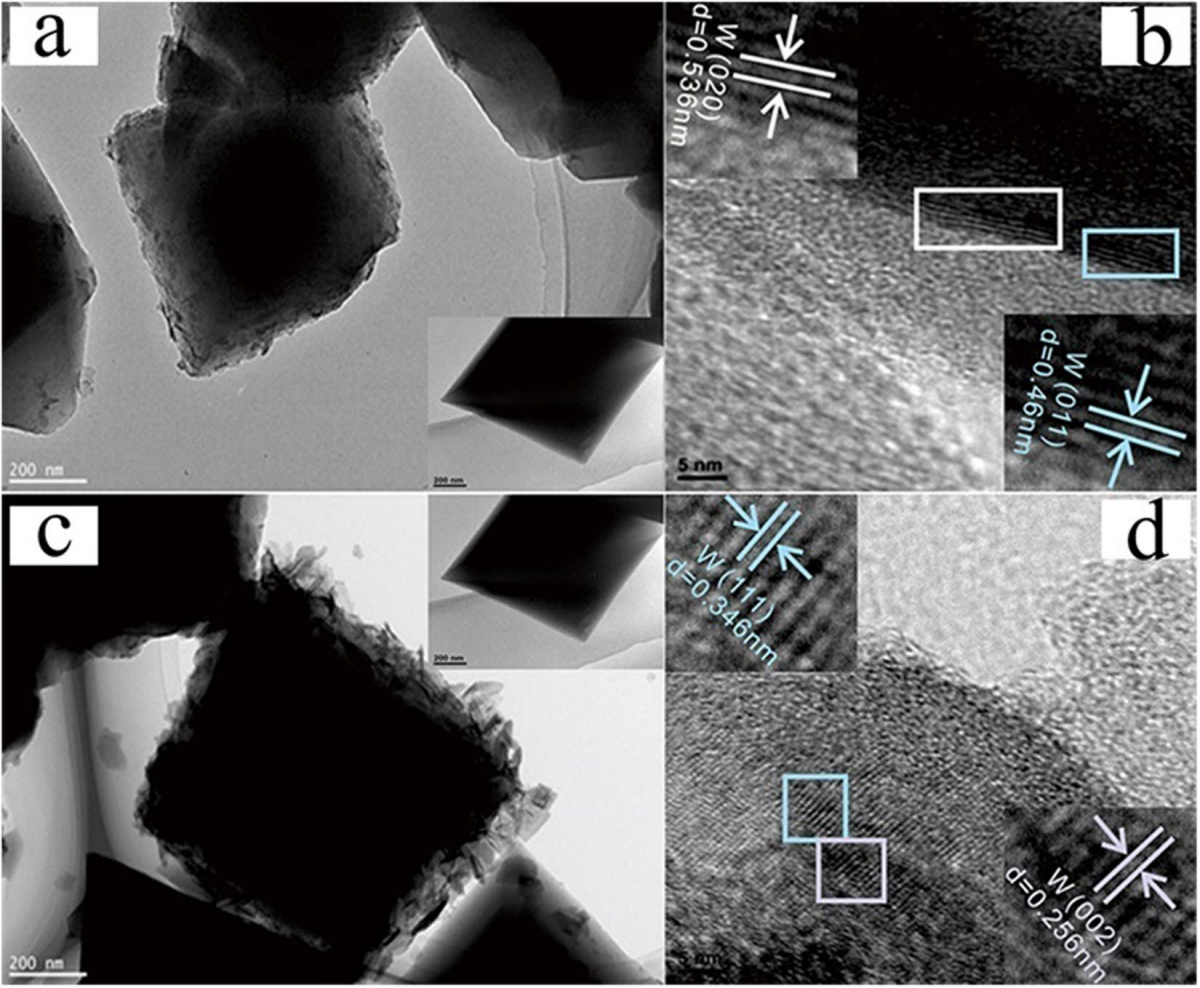
(**a**) TEM image of (10%) WO_3_@MIL-101@WO_3_ (insert was pure MIL-101), (**b**) HRTEM image of (10%) WO_3_@MIL-101@WO_3_ and the reflection of crystal face about its inverse FFT, (**c**) TEM image of (10%) WO_3_&MIL-101 (insert was pure MIL-101), (**d**) HRTEM image of (10%) WO_3_&MIL-101 and the reflection of crystal face about its inverse FFT.

In order to study the morphology of MIL-101 (Fig. 5a,c insert), TEM and HRTEM measurements were carried^27^. The images of WO_3_@MIL-101@WO_3_ and WO_3_&MIL-101, MIL-101, WO_3_ were shown in Fig. 5. The insert picture shows a typical octahedral structure of MIL-101 which have an average size of 500 nm^27^. In the WO_3_@MIL-101@WO_3_ sample, WO_3_ was very little and well coating outside MIL-101, in WO_3_&MIL-101 sample, WO_3_ nanoparticles with a small size are well dispersed outside of MIL-101 without obvious aggregation, demonstrating that the MIL-101 can well hindering the growth of WO_3_ as an excellent matrix^27^. Displayed by the HRTEM images of WO_3_@MIL-101@WO_3_ and WO_3_&MIL-101, the marked lattice pitch of 0.536 nm and 0.46 nm on the surface of MIL-101 is corresponded to the (020) and (011) planes of WO_3_, and the marked lattice pitch of 0.346 nm and 0.256 nm on the shell is matched with the (111) and (002) planes of WO_3_. These results suggest that an intimate contact which will be helpful for the charge separation and transferring between WO_3_ and MIL-101. The energy dispersive X-ray spectroscopy (EDS) mapping (Fig. S5) was conducted to further confirmed the component and structure of WO_3_@MIL-101@WO_3_, the crystal structure of MIL-101 can be displayed by the uniform Cr elements in the background^27^.

**Figure 6:**
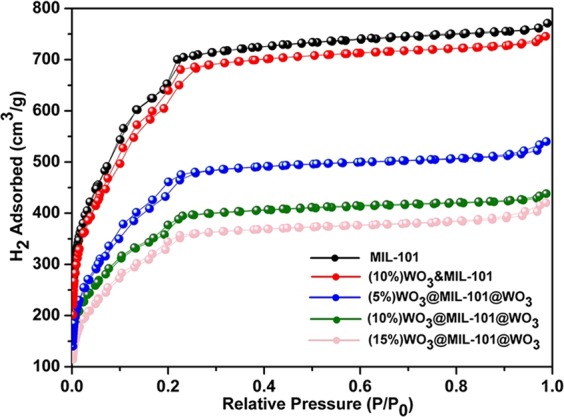
BET adsorption–desorption isotherm of MIL-101, (10%) WO_3_&MIL-101 and different loading percentage of WO_3_@MIL-101@WO_3_.

To clarify the location of WO_3_ relative to MIL-101, the N_2_ adsorption measurement of MIL-101 was carried which shows type I property with secondary uptakes at p/p_0_ of around 0.1 and 0.2 (Fig. 6a), which is a typical MIL-101 adsorption curve^50,51^. After loading WO_3_ nanoparticles, the adsorption-desorption isotherm of WO_3_&MIL-101 sample has little changes but the adsorption-desorption isotherm of WO_3_@MIL-101@WO_3_ has a significant change compared with MIL-101 and WO_3_&MIL-101 and N_2_ adsorption decreased with the WO_3_ content increased. The surface area of MIL-101 was measured to be 2480 m^2^/g and the total pore size value was estimated to be 1.193 cm^3^/g at a relative pressure of 0.99 (shown in Table 1), both are similar to the numbers reported in the literature. The surface area of WO_3_&MIL-101 was 2350 m^2^/g and pore size value was 1.153 cm^3^/g, both are close to MIL-101. The surface area of WO_3_@MIL-101@WO_3_ samples gradually decreased from 1668 to 1255 m^2^/g, the pore size value change from 0.835 to 0.651 cm^3^/g, as WO_3_ content increased from 5% to 15%. The pore size distribution is shown in Fig. S7, the pore size at 1.8, 2.6 and 3.2 nm are attributed to pure MIL-101 which has been reported in literature^50^, compared with pure MIL-101, the pores size distribution of the WO_3_&MIL-101 have little changes while the pore size of WO_3_@MIL-101@WO_3_ have a significantly decrease than both pure MIL-101 and WO_3_&MIL-101. With the WO_3_ content increased, the pore size shows a decreasing trend. All these phenomena could be caused by WO_3_ embed into the pores of MIL-101. These results suggest that the WO_3_ nanoparticles in WO_3_@MIL-101@WO_3_ samples are successfully embedded in the cavities of MIL-101. WO_3_ nanoparticle has little influence for surface area and pore size of MIL-101, so WO_3_ nanoparticles are possibly on the surface of MIL-101 in WO_3_&MIL-101 sample.

**Table 1:** The surface area and pore size volume of MIL-101, (10%) WO_3_&MIL-101 and different loading percentage of WO_3_@MIL-101@WO_3_.

Sample	BET Surface area (m^2^/g)	Pore size Volume (cm^3^/g)
MIL-101	2480	1.193
(10%) WO_3_&MIL-101	2350	1.153
(5%) WO_3_@MIL-101@WO_3_	1668	0.835
(10%) WO_3_@MIL-101@WO_3_	1360	0.678
(15%) WO_3_@MIL-101@WO_3_	1255	0.651

Combined with the TEM and SEM results, it can be concluded the WO_3_ particles just coat on the surface of MIL-101 in WO_3_&MIL-101 sample. The WO_3_ particles partly embed into the pores and partly on the surface of MIL-101 in WO_3_@MIL-101@WO_3._

### UV-Vis DRS analysis

**Figure 7:**
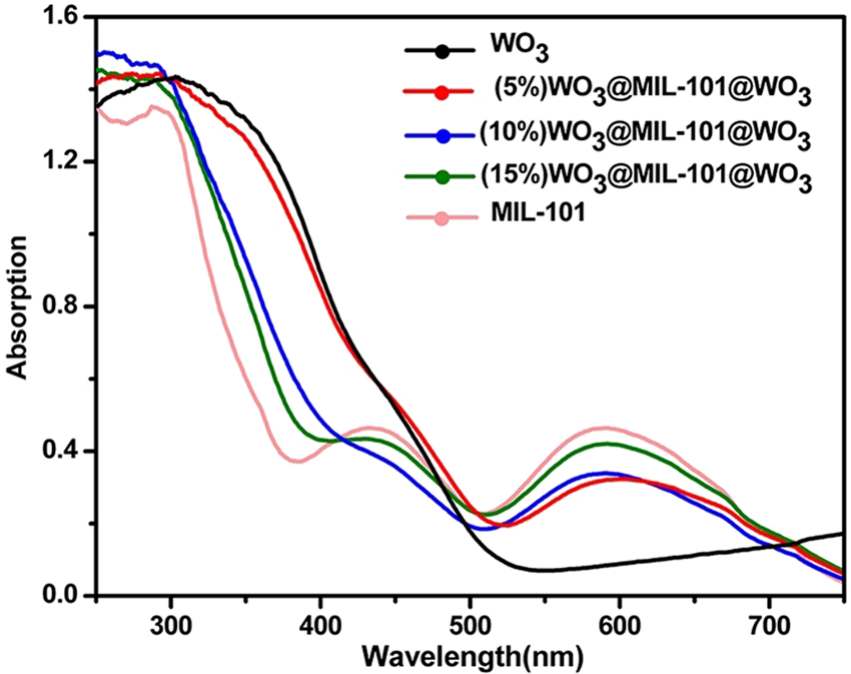
UV–vis DRS spectra of WO_3_, different loading percentage of WO_3_@MIL-101@WO_3_ and MIL-101.

UV-Vis DRS spectra were used to analyze the optical properties of the MIL-101 and the different WO_3_ loading proportion samples (Fig. 7). MIL-101 exhibits two characteristic absorption band centered at 450 nm and 600 nm, which coincides with that in literature^27^. The band of pure MIL-101 in the UV region belongs to π-π* transitions of ligands and the bands in the visible region can be assigned to the D-D spin-allowed transition of the Cr^3+^. WO_3_ displays a sharp fundamental absorption edge rise at 475 nm as expected, corresponding to a band gap of 2.75 eV^7^. Compared with that of MIL-101, the WO_3_@MIL-101@WO_3_ shows an enhanced board absorption in visible light region, this may correspond to the visible light enhanced of WO_3_. Compared with that of pure WO_3_, WO_3_@MIL-101@WO_3_ composite shows an adsorption band centered at 600 nm, which is attributed to the absorption of MIL-101 matrix.

### Photocatalytic Degradation of Organic Pollutant

**Figure 8:**
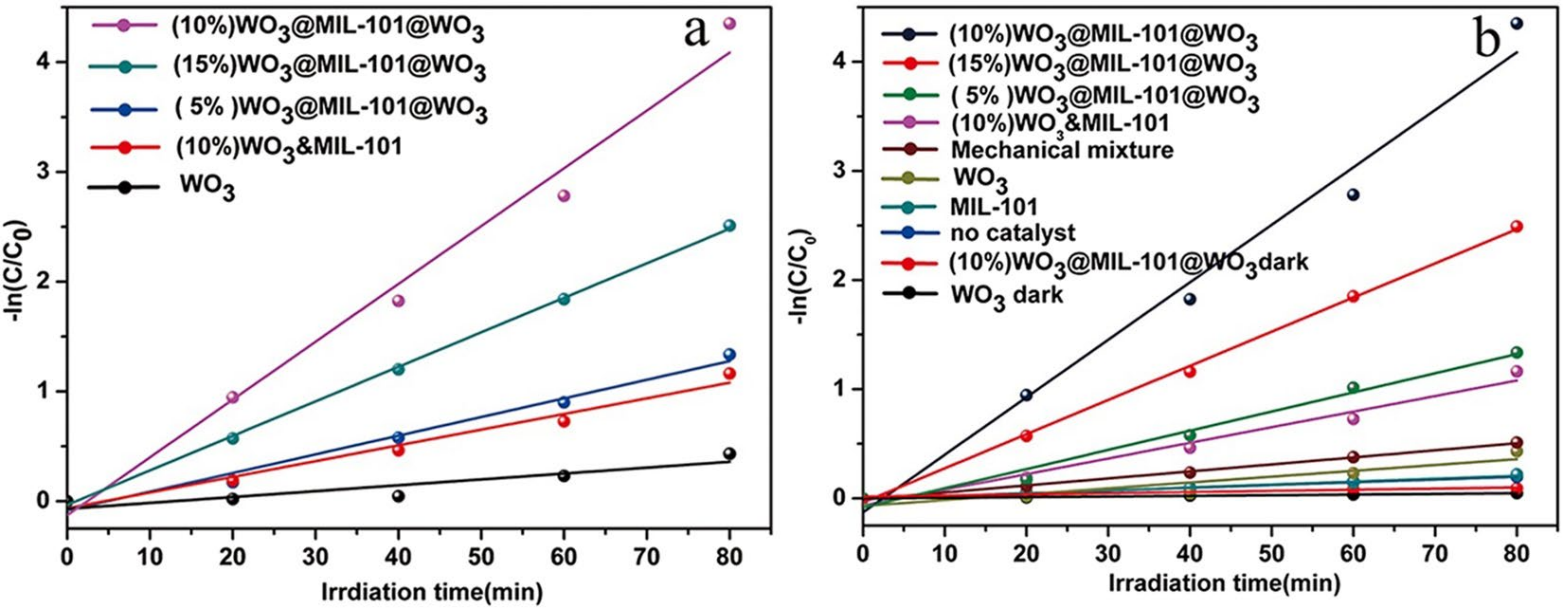
(**a**) The reaction rate constants (k) of different loading percentage WO_3_@MIL-101@WO_3_, (10%)WO_3_&MIL-101 and WO_3_. (**b**) The reaction rate constants (k) of samples with different loading percentage and a series of control experiments.

The MIL-101 loading WO_3_ samples were evaluated for photocatalytic MB degradation. As shown in Fig. 8a, comparing with pure WO_3_, the embedded structure WO_3_@MIL-101@WO_3_ and coating structure WO_3_&MIL-101 increased 9 times and 3 times, respectively, due to the closely contact between WO_3_ and MIL-101 which can be concluded from XPS and DLS data. Also, the embedded structure has 3 times higher efficiency than coating structure due to the part of WO_3_ have embeded into the pores of MIL-101 resulting in the shorter distance of the electrons transfer from WO_3_ to MIL-101 comparing with coating structure^52^. Figure 8b show the degradation efficiency of different loading percentages and a series of control experiment, which shown 10% WO_3_ loading sample has the best photocatalytic efficiency. From the control experiment, it can be seen that pure MIL-101 has no photocatalytic efficiency, MB cannot be degraded by self-sensitization and the light are the necessary condition during photocatalytic. Different pH and concentration were also investigated in Fig. S8a,b which shown that pH and concentration influence the photocatalytic efficiency of MB degradation. The WO_3_@MIL-101@WO_3_ has the best photocatalytic efficiency when the amount of HCl was 280 μL and the volume of water was 35 mL.

### Mechanism Investigation on Photocatalytic Performance Improvement

**Figure 9:**
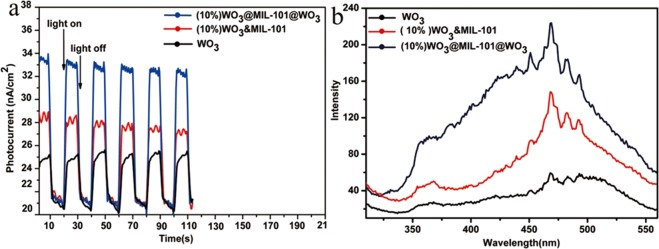
(**a**) Photocurrent responses of WO_3_, (10%) WO_3_&MIL-101 and (10%) WO_3_@MIL-101@WO_3_, (**b**) PL spectra of WO_3_, (10%) WO_3_&MIL-101 and (10%) WO_3_@MIL-101@WO_3_.

To further unveil the higher photocatalytic efficiency of WO_3_@MIL-101@WO_3_ than WO_3_&MIL-101, photocurrent measurement have been carried and the results show that the photocurrent for both MIL-101 supported WO_3_ get enhanced as compared to the pristine WO_3_ (Fig. 9a), revealing that the formation of WO_3_-MOF schottky junction helps to separate the photo-generated electron-hole pairs^52^. The WO_3_@MIL-101@WO_3_ displays much stronger photocurrent response than WO_3_&MIL-101, suggesting the much higher efficiency of the charge transfer^52^. This result is also supported by the photoluminescence (PL) emission spectroscopy, which provides useful hints for the photo-excited charge transfer and recombination. The PL intensity is slightly weakened when the WO_3_ only coating outside MOF, while get greatly suppressed when some WO_3_ nanoparticles have dispersed inside the MOF (Fig. 9b). These observations indicate that the irradiative electron-hole recombination is more effectively suppressed by extracting the electrons from internal WO_3_ than coating WO_3_ ^53^. Such distinctly different photoelectron-chemical properties in WO_3_@MIL-101@WO_3_ and WO_3_&MIL-101 unambiguously demonstrate that the part of WO_3_ in the pores of MIL-101 contribute mostly of the photocatalytic efficiency of MB degradation. For comparison, we also investigated the photoluminescence (PL) emission spectroscopy of different WO_3_ loading percentage (Fig. S9), the PL intensity are corresponding with the photocatalytic efficiency. The WO_3_ loaded MOF samples all have lower intensity than pure WO_3_, pure MIL-101 have a lower photoluminescence emission. It indicated that WO_3_-MOF schottky junction can well suppressed the electron-hole pairs recombination and pure MIL-101 cannot be excited by visible light. In order to further investigate the migration and interface transfer or recombination rates of charge carriers electrochemical impedance spectra (EIS) was detected in Fig. S10. It was found that the WO_3_@MIL-101@WO_3_ and WO_3_&MIL-101 composite exhibits much smaller arc sizes than the pure WO_3_ under visible light irradiation. It demonstrates that the heterojunction composite has faster electron transfer through an intimated interface between MIL-101 and WO_3_ as compared to the pristine WO_3_, which is in good agreement with the photocatalytic performance.

**Figure 10:**
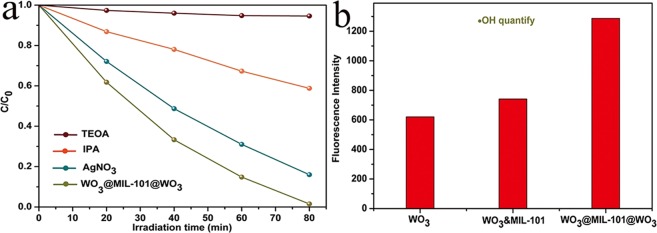
(**a**) Trapping experiment of WO_3_@MIL-101@WO_3_ with IPA, AgNO_3_, TEOA and no quenching. (**b**) Transformation percentage of TA by WO_3_, WO_3_&MIL-101 and WO_3_@MIL-101@WO_3_.

The photocatalytic mechanism of WO_3_@MIL-101@WO_3_ have been researched by active species trapping and ·OH quantify experiment during the photocatalytic process^48^. In order to study the active species of the photocatalytic reaction of WO_3_@MIL-101@WO_3_, the trapping experiment was investigated and showed in Fig. 10a. It can be concluded that the addition of AgNO_3_ (a quencher of e-, which can hinder the formation of O^2−^·) have no influence on photocatalytic degradation of MB^48^. On the contrary, the addition of IPA (a quencher of ·OH) or TEOA (a quencher of h^+^) have an obvious influence of decrease on the photocatalytic degradation of MB. Therefore, the conclusion can be drawn that photo-generated holes (h^+^) and ·OH are the main effective species on MB degradation for WO_3_@MIL-101@WO_3_ under visible light irradiation. The result consistent with the kind of effective species of pure WO_3_ and WO_3_&MIL-101. It can be concluded that the kind of active species have not changed after the combined. ·OH production quantification experiments have been revealed by the fluorescent intensity of TAOH for WO_3_, WO_3_&MIL-101 and WO_3_@MIL-101@WO_3_ in Fig. 10b. For WO_3_&MIL-101, the produce of ·OH just have little changed compared with pure WO_3_. But for WO_3_@MIL-101@WO_3_ the fluorescent intensity of TAOH compared with pure WO_3_ were totally different, there has a significant increase of ·OH. Above result can be explained: the electron produced from WO_3_ conduction band can be easily recombined due to the positive conduction level because of rapidly recombination of electron-hole pairs, so there are little ·OH produced for WO_3_. For WO_3_&MIL-101, because of the similar ·OH quantify result and the result of PL and photocurrent, the mechanism is the same as pure WO_3_. This can be explained that WO_3_ coating outside of MIL-101 only, nanoparticles are tending to be aggregating and electrons are favoring to be stacking and recombination rate increase. For WO_3_@MIL-101@WO_3_ the electrons produced from conduction band of WO_3_ transferred to MIL-101 due to the shorter electrons transfer distance. Due to the transformation of electrons, the holes can be separated to a greater degree, as a result, ·OH can be easily produced from high valence position of h^+^.

**Figure 11:**
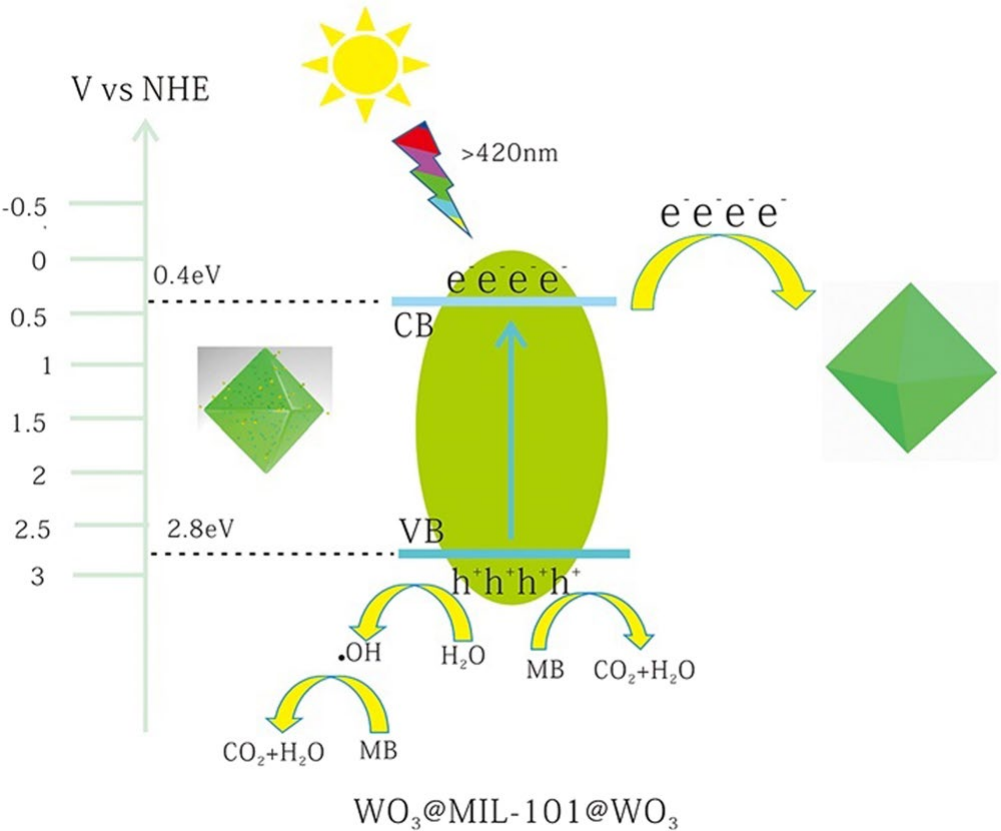
The possible photocatalytic mechanism scheme of WO_3_@MIL-101@WO_3_ under visible light irradiation (λ ≥ 420 nm).

**Figure 12:**
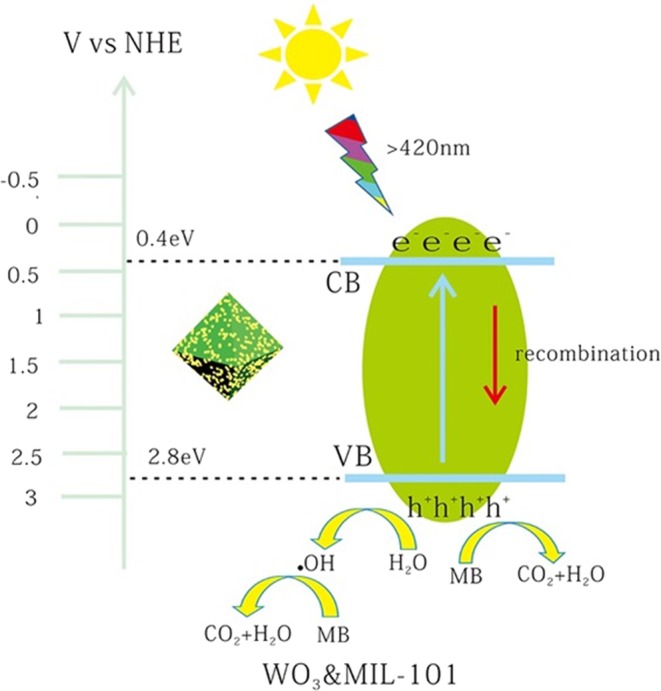
The possible photocatalytic mechanism scheme of WO_3_&MIL-101 under visible light irradiation (λ ≥ 420 nm).

This may also conclude that WO_3_ in the pores of MIL-101 have a shorter distance to transfer electrons from WO_3_ conduction band resulting in higher electrons-hole separation efficiency comparing with WO_3_&MIL-101, the same conclusion have been shown in the literature^52^. It may be the reason the embedded structure has higher efficiency than coating structure. The possible photocatalytic mechanism of WO_3_@MIL-101@WO_3_ and WO_3_&MIL-101 were shown in Figs 11 and 12.

## Conclusion

The WO_3_@MIL-101@WO_3_ and WO_3_&MIL-101 hetero-structure were successfully synthesized, the WO_3_ nanoparticles were successfully embedded into the pores of MIL-101. MIL-101 can confine the particle size of WO_3_ and prevent the nanoparticles from aggregation and leaching to result in the enhancement of photo activity. Different loading percentage, pH and concentration were investigated to boost the photocatalytic degradation of MB, a great enhancement in photocatalytic activity is 10% content embedded structure sample, which increased 9 times activity compared with pure WO_3_ and 3 times compared with WO_3_&MIL-101. The photocatalytic mechanism of WO_3_@MIL-101@WO_3_ were investigated. Photocurrent, PL and ·OH, quantify experiment all show that when WO_3_ embedded into the pores of MIL-101, MIL-101 can play the role of promoting charge separation due to the short distance improving the electron-hole separation efficiency. The synthesis strategy presented here can be expended as a facile approach to synthesizing related dipping metal oxide into the pores of metal-organic framework for functional design and application.

## Supplementary information

Supplementary Material of the manuscript
