# Supplementary material for: WO3 in suit embed into MIL-101 for enhancement charge carrier separation of photocatalyst
Source: Sci Rep. 2019 Mar 19;9:4860. doi: 10.1038/s41598-019-41374-z (PMC6425017; doi:10.1038/s41598-019-41374-z)
Supplement: Supplementary file 1 — Supplementary Material of the manuscript [file 41598_2019_41374_MOESM1_ESM.pdf]

WO_3 in suit embed into MIL-101 for enhancement charge carrier separation of photocatalyst

Linjuan Wang, Ling Zan*
College of Chemistry and Molecular Science, Wuhan University, Wuhan 430072, P. R. China.
E-mail: irlab@whu.edu.cn(L. Zan) Tel: 86 27 6875 2919

Table S1. Different condition of the prepared samples

Sample name	$Na_2WO_4 \cdot H_2O$ quality (mg)	HCl volume (μL)	H_2O volume (mL)	H_2O_2 volume (μL)	t(h)	$\eta = k/k_{WO3}$
(5%)WO_3@MIL-101@WO_3	5	320	40	100	24	2.7
(10%)WO_3@MIL-101@WO_3	10	320	40	100	24	9.8
(10%)WO_3&MIL-101	10	320	40	100	0.5	3.17
(15%)WO_3@MIL-101@WO_3	15	320	40	100	24	3.83
No hydrogen peroxide	10	320	40	0	24	/
H_{HCl160}	10	160	40	100	24	3.96
H_{HCl280}	10	280	40	100	24	8.76
H_{HCl480}	10	480	40	100	24	7.74
H_{HCl640}	10	640	40	100	24	5.53
C_{W20}	10	320	20	100	24	3
C_{W30}	10	320	30	100	24	4.6
C_{W45}	10	320	45	100	24	7.36
C_{W50}	10	320	50	100	24	1.8
WO_3	500	1000	40	200	6h	1

Under different condition the peroxy tungsten gel will have different concentration and state, the loading percentage, pH, and concentration are the factors of the influence of the condition. The reaction conditions are listed in **Table S1**.

Table S2. The Atomic Absorption Spectroscopy (AAS) of samples with different loading percentages

Sample	(5%)WO_3@MIL-101@WO_3	(10%)WO_3@MIL-101@WO_3	(15%)WO_3@MIL-101@WO_3	(10%)WO_3&MIL-101
Theoretical percentage	5%	10%	15%	10%
Reality percentage	5.27%	10.23%	15.5%	10.25%

The atomic absorption spectroscopy was conducted to exam the real content of the loading percentage (**Table S2**) compared with theoretical percentage, the results showed that the reality percentages consistent with the theoretical percentage.

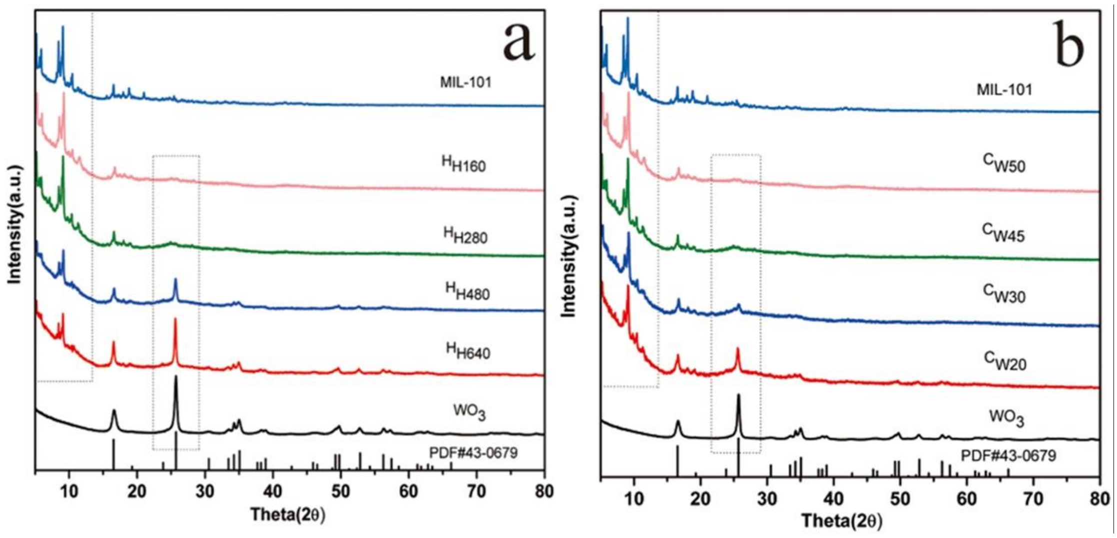

Figure S1. (a) The XRD patterns of samples with different pH changed by HCl volume, (b) The XRD patterns of samples with different concentration changed by the water of sodium tungstate

Table S3: Zeta potential of WO_3, (10%)WO_3&MIL-101, (10%)WO_3@MIL-101@WO_3 and MIL-101

Samples	WO_3	(10%)WO_3&MIL-101	(10%)WO_3@MIL-101@WO_3	MIL-101
ZP-Ave (mv)	-34.1	-8.69	-6.04	34.5

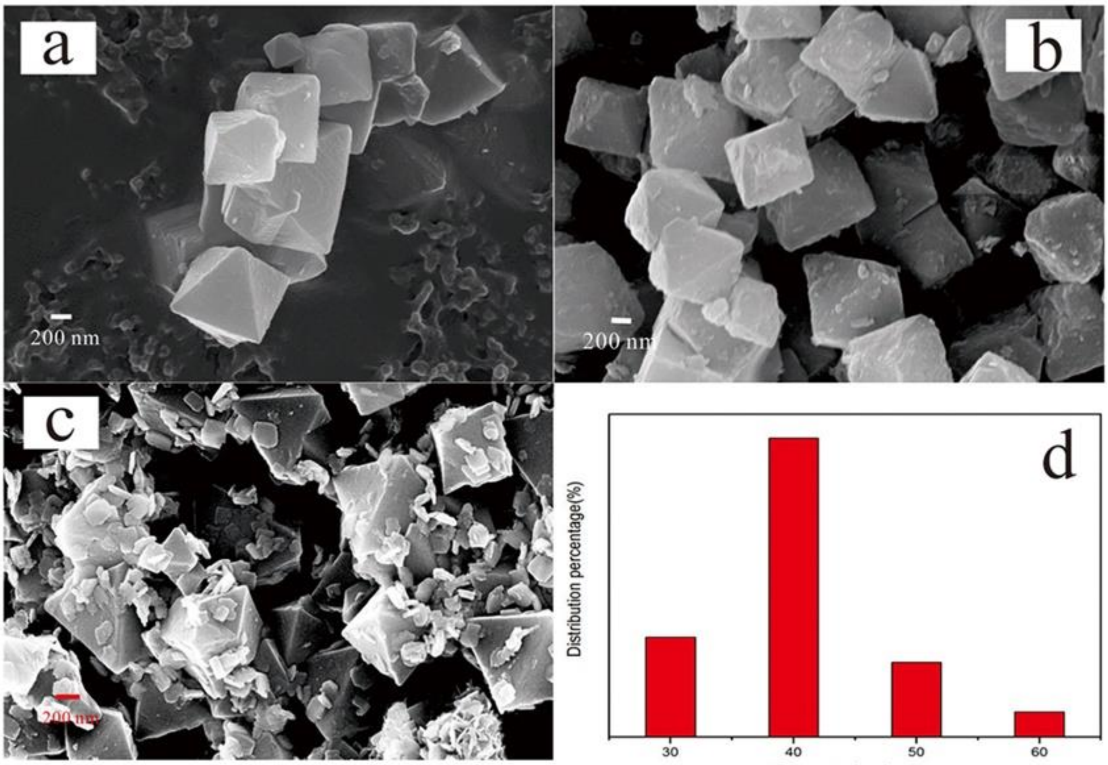

Figure S2. SEM images of (a) (5%)WO_3@MIL-101@WO_3, (b) (15%)WO_3@MIL-101@WO_3, (c) No hydrogen peroxide, (d) The particle size distribution of (10%)WO_3&MIL-101

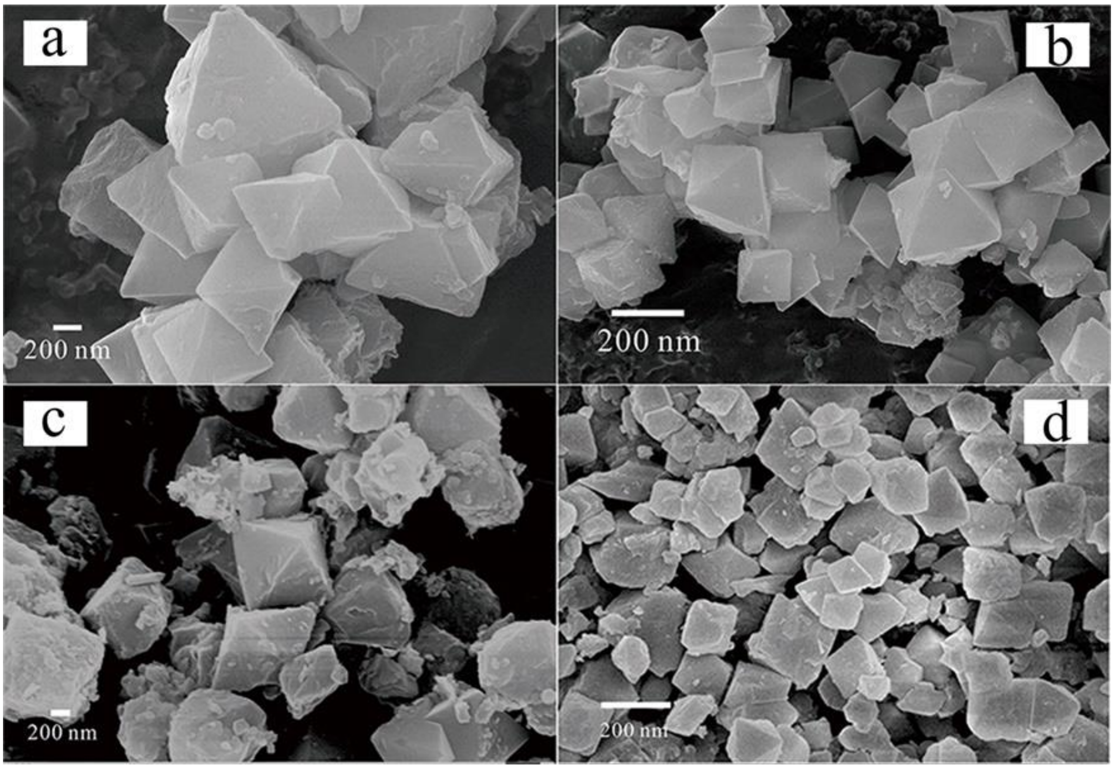

Figure S3. The surface morphpoly of samples: (a) H_{HCl160}, (b) H_{HCl280}, (c) H_{HCl480}, (d) H_{HCl640}

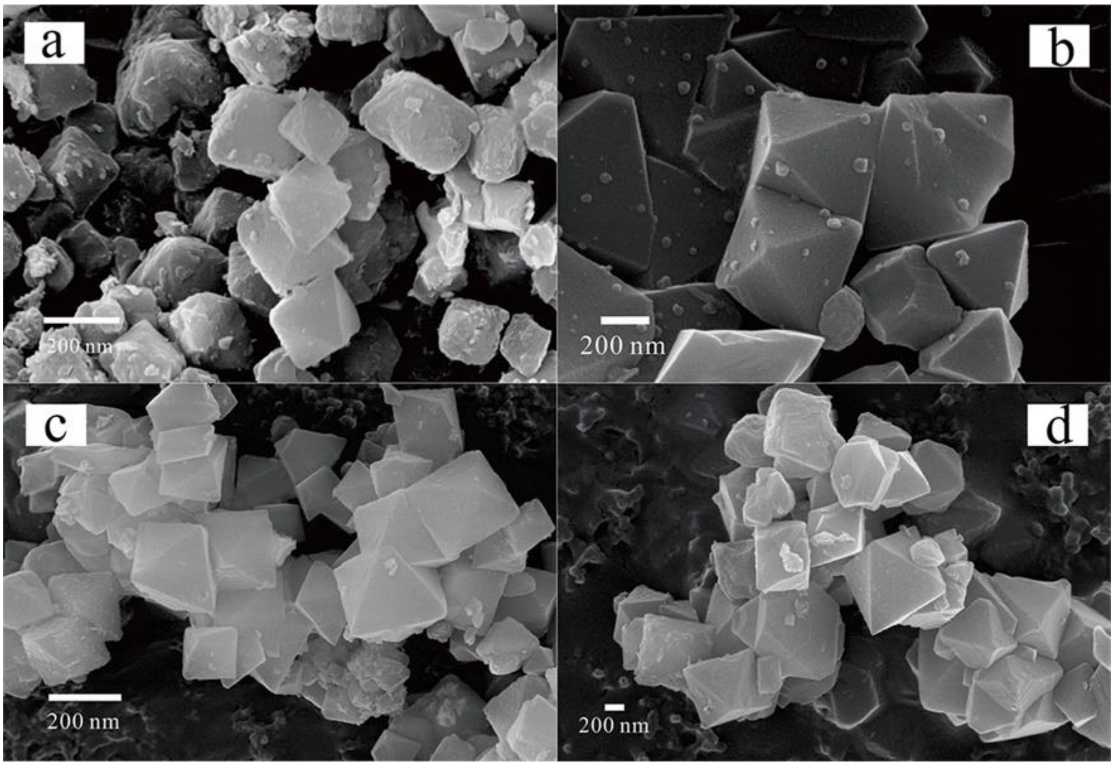

Figure S4. The surface morphpoly of samples: (a) C_{W20}, (b) C_{W30}, (c) C_{W45}, (d) C_{W50}

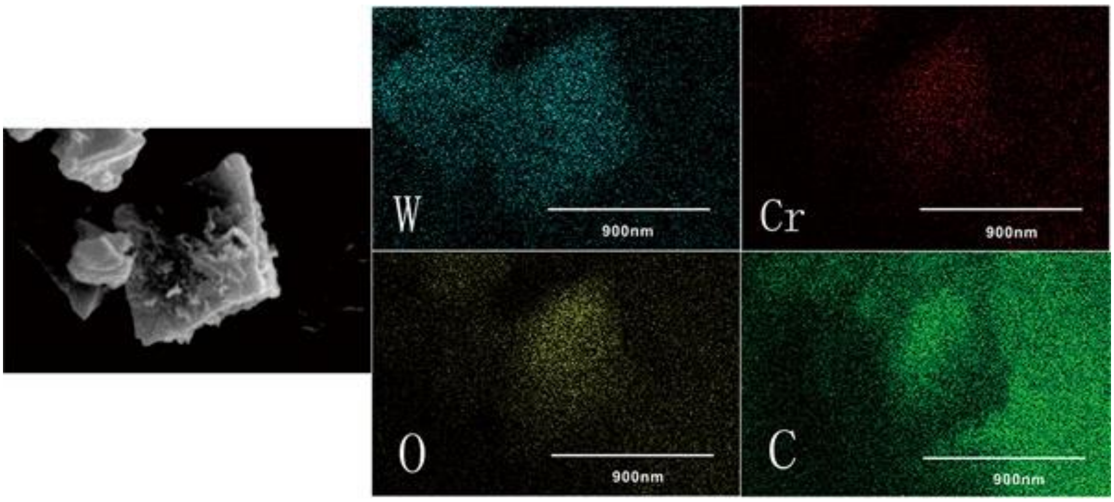

Figure S5. EDS Mapping of (10%)WO_3@MIL-101@WO_3

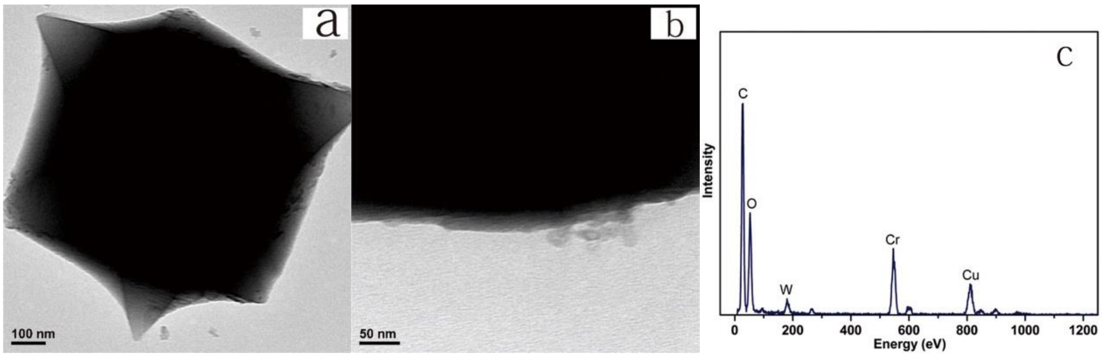

Figure S6. (a,b) The TEM images of (5%)WO_3@MIL-101@WO_3, (c) EDX analysis result of (5%)WO_3@MIL-101@WO_3

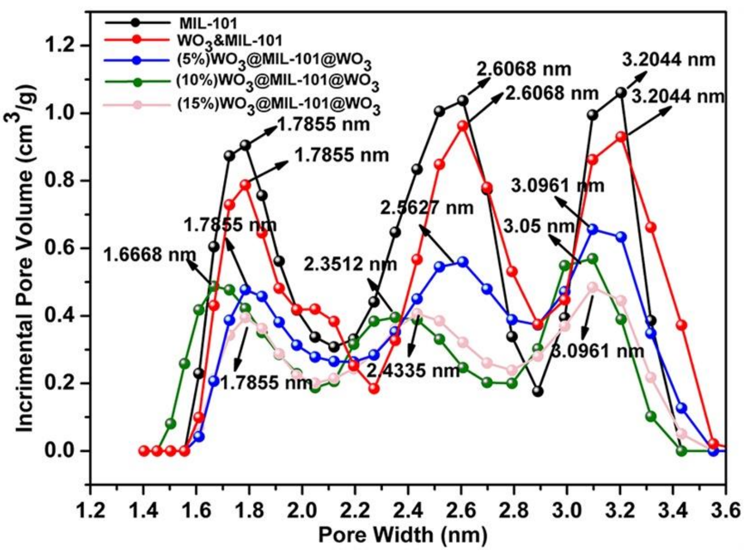

Figure S7. BET pore size distribution of different loading percentage.

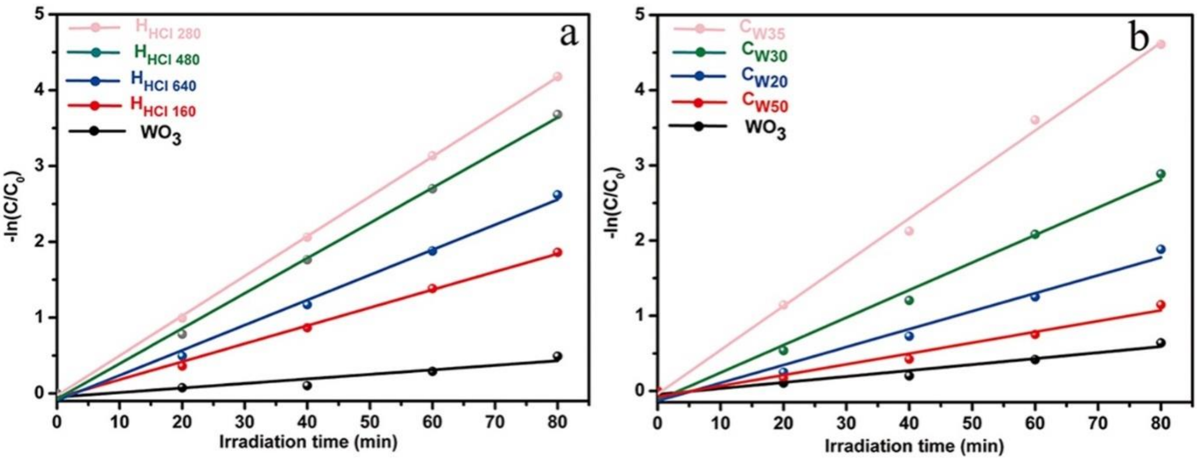

Figure S8. (a) The reaction rate constants (k) of samples with different pH on the degradation of MB, (b) The reaction rate constants (k) of samples with different concentration on the degradation of MB.

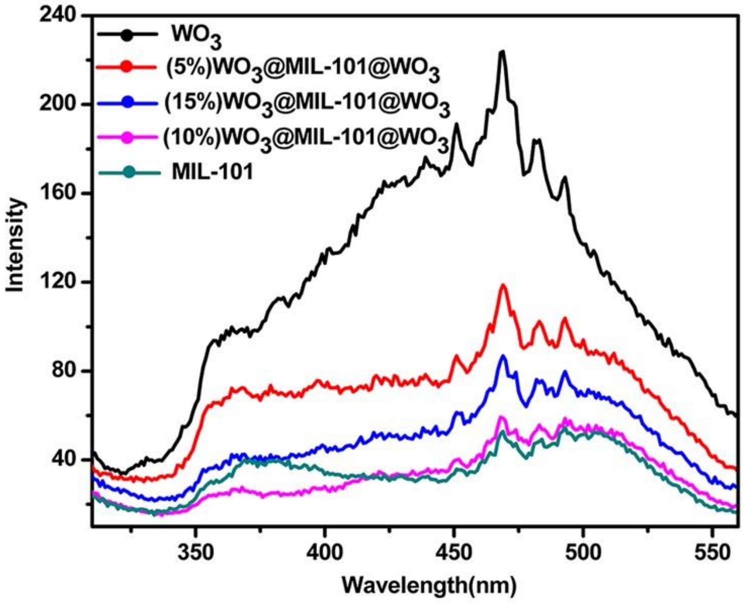

Figure S9. PL spectra of WO_3, (5%)WO_3@MIL-101@WO_3, (10%)WO_3@MIL-101@WO_3, (15%)WO_3@MIL-101@WO_3 and MIL-101.

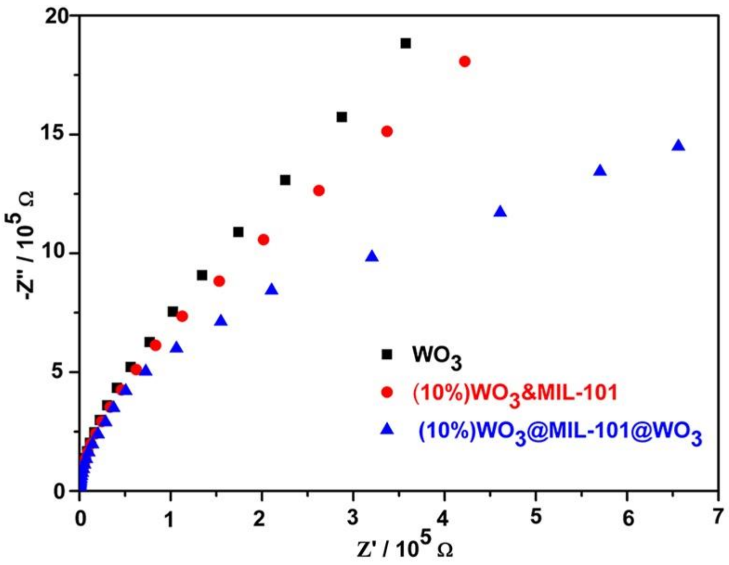

Figure S10. EIS Nyquist plots of WO_3, (10%)WO_3&MIL-101 and (10%)WO_3@MIL-101@WO_3 under visible light irradiation
